# Ceftazidime/avibactam combined with colistimethate sodium successfully cures carbapenem-resistant *Pseudomonas aeruginosa*-induced brain abscess in a child post-craniotomy: a case report

**DOI:** 10.3389/fonc.2024.1444172

**Published:** 2024-09-19

**Authors:** Minglu Yuan, Miao Zong, Cong Ren, Wenjing Zong, Zhongdong Li

**Affiliations:** ^1^ Department of Pharmacy, Beijing Electric Power Hospital of State Grid Co. of China, Capital Medical University Electric Teaching Hospital, Beijing, China; ^2^ Department of Neurosurgery, Beijing Electric Power Hospital of State Grid Co. of China, Capital Medical University Electric Teaching Hospital, Beijing, China; ^3^ Institute of Chinese Materia Medica, China Academy of Chinese Medical Sciences, Beijing, China

**Keywords:** brain abscess, central nervous system infections, carbapenem-resistant pseudomonas aeruginosa, ceftazidime/avibactam, colistimethate sodium

## Abstract

The treatment of brain abscess induced by carbapenem-resistant *Pseudomonas aeruginosa* (CRPA) is a clinical challenge around the world. Apart from novel β-lactam/β-lactamase inhibitors and polymyxins, there are few sufficiently powerful antibiotics that are effective against CRPA-induced infections. Considering the blood-brain barrier factor, there are even fewer drugs that can be used to treat intracranial CRPA-induced infections. In this article, we reported a case of CRPA-induced brain abscess that was successfully treated with intravenous ceftazidime/avibactam and intrathecal colistimethate sodium in a child after intracranial tumor resection.

## 1 Introduction

Brain abscess is a serious postoperative complication after neurosurgical craniectomy, which usually leads to prolonged hospitalization and higher mortality rates. Although the incidence is only 0.17% (1), once a brain abscess has formed, more than 60% of patients need secondary craniectomy due to the limited availability of effective antibiotics (2). Brain abscesses are mainly caused by Gram-positive cocci, but 20% are due to Gram-negative bacilli (2, 3). *Pseudomonas aeruginosa* (PA), which is highly virulent and invasive, is one such Gram-negative bacillus. The risk factors for PA-induced central nervous system infections (CNSI) include craniocerebral trauma, otorhinolaryngology-head and neck surgery, cerebrospinal fluid (CSF) leakage or ventricular drainage, implantation of invasive catheter, spreading of ear or sinus infections, immune deficiency, and chemotherapy. If CNSI is caused by CRPA, clinical treatment is extremely difficult.

Traditional antibiotics with anti-PA activity include piperacillin/tazobactam, cefoperazone/sulbactam, ceftazidime, aztreonam, quinolones (ciprofloxacin, levofloxacin), aminoglycosides (gentamicin, amikacin), and carbapenems (meropenem, imipenem). If CRPA is susceptible to these conventional antibiotics, it should be treated with them in the first instance. However, if these drugs do not effectively control the CRPA-induced infection or the infection is severe, a novel β-lactam/β-lactamase inhibitor can be used, such as ceftolozane/tazobactam, ceftazidime/avibactam, imipenem-cilastatin/relebactam, meropenem/vaborbactam, or polymyxins combined with other conventional sensitive drugs (4). In addition, some studies reported that a combination of antibiotics with glucocorticoids or vitamin C could effectively reduce the brain abscess area by modulating glucocorticoid receptor and toll-like receptor-2 expression, glucocorticoids or vitamin C can be considered as an adjuvant therapy (5–7). Because polymyxins such as colistimethate sodium (CMS) barely penetrate the blood-brain barrier (BBB), CMS has been approved for ventricular/intrathecal administration to treat CRPA-induced intracranial infections. In addition, among these novel β-lactam/β-lactamase inhibitors, only ceftazidime/avibactam, an excellent antimicrobial drug against most carbapenem-resistant Enterobacteriaceae (CRE), including CRPA (8), has been approved for clinical application in China. Some studies have reported that ceftazidime/avibactam is able to penetrate the BBB to treat intracranial infections, but the degree of penetration into the brain abscess is still unknown. Currently, intravenous ceftazidime/avibactam combined with intrathecal CMS, or intravenous or intrathecal amikacin, or intravenous fosfomycin have been reported to successfully treat CRPA-induced meningitis or ventriculitis (8–13), but the use of these drugs for treatment of CRPA-induced brain abscess has not yet been reported. In this article, we presented the first case of a child with CRPA-induced brain abscess after intracranial tumor resection that has been successfully cured by intravenous ceftazidime/avibactam combined with intrathecal CMS. We have also reviewed the literature to provide new ideas and experience to inform clinical pharmacological therapy of CRPA-induced brain abscess.

## 2 Case report

The male patient was 14-years-old, 168 cm tall, and weighed 55 kg. He had complained of intermittent headaches and vomiting since September 2023. A ventricular tumor was identified by enhanced magnetic resonance imaging (MRI) and the patient underwent ventricular tumor resection by ventriculoscope at another tertiary care hospital on October 4th 2023. The pathological type of the tumor was subventricular giant cell astrocytoma. The patient developed positive cervical resistance immediately after surgery, with a maximum body temperature of 39°C. He was treated by intermittent lumbar puncture and a combined regimen of linezolid and meropenem for 20 days. Unfortunately, the treatment had little success. Therefore, the patient was transferred to our hospital for further treatment.

On day 1, he had a body temperature of 39°C with nuchal rigidity. He underwent lumbar cistern drainage, which drained-out turbid orange CSF. Routine and biochemical examination of the CSF showed a positive Pandy test with a leukocyte count of 192/mm^3^ (normal range: 0-8/mm^3^), microprotein 273 mg/dL (normal range: 0-40 mg/dL), chloride 118.6 mmol/L (normal range: 120-132 mmol/L), and glucose 2.64 mmol/L (normal range: 2.5-4.4 mmol/L). A computed tomography (CT) scan of the head showed slight hyperdensity along the surgical path. Vancomycin (20 mg, qd, intrathecal) combined with meropenem (2 g, q8h, intravenous) was used empirically. On day 4, the child remained intermittently febrile with a maximum body temperature of 40°C. Accordingly, the CSF was sampled for bacterial culture and drug susceptibility testing.

**Figure 1:**
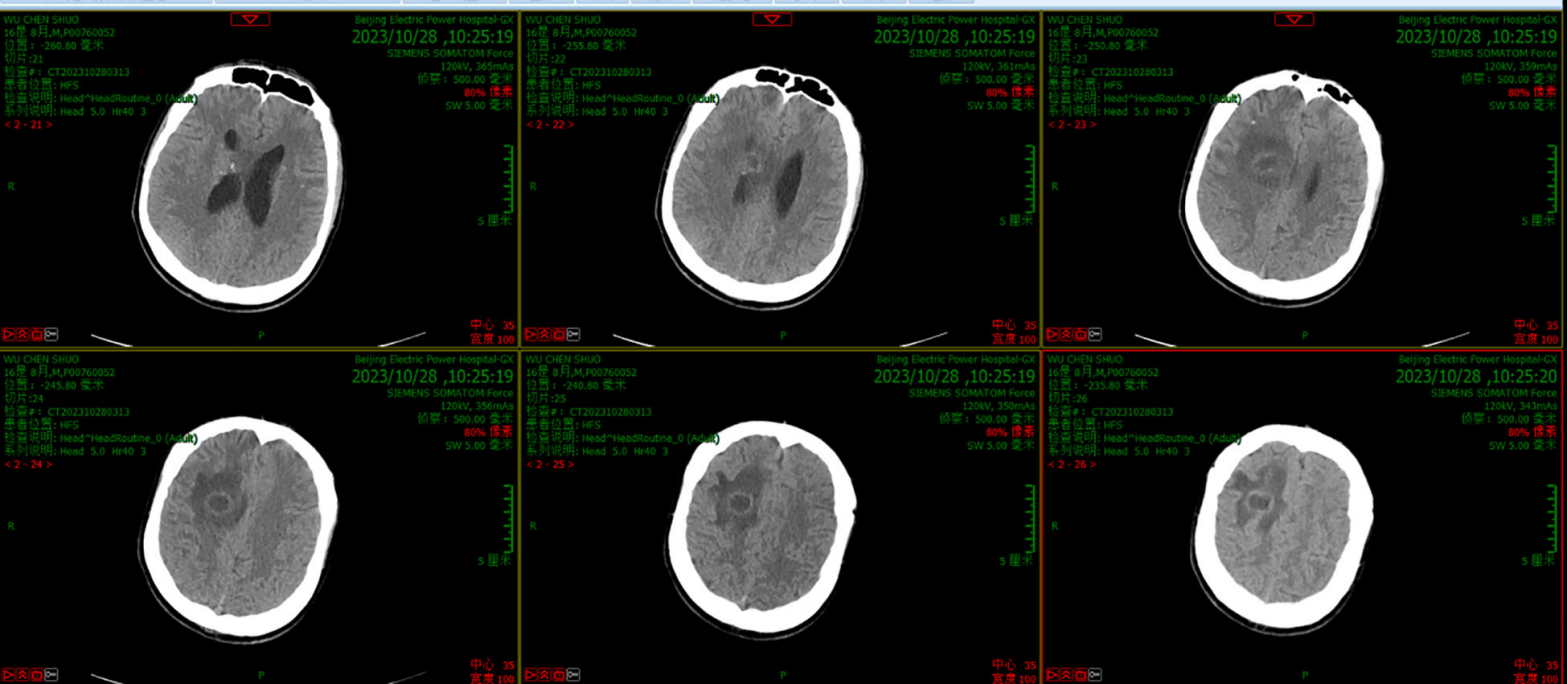
CT scanning before the new therapeutic regimen showed a round cystic-solid lesion in the operative area, with the interior of the lesion being hypointense and the surrounding solid cystic wall being hyperintense. The brain tissue around the lesion was evidently edematous.

**Figure 2:**
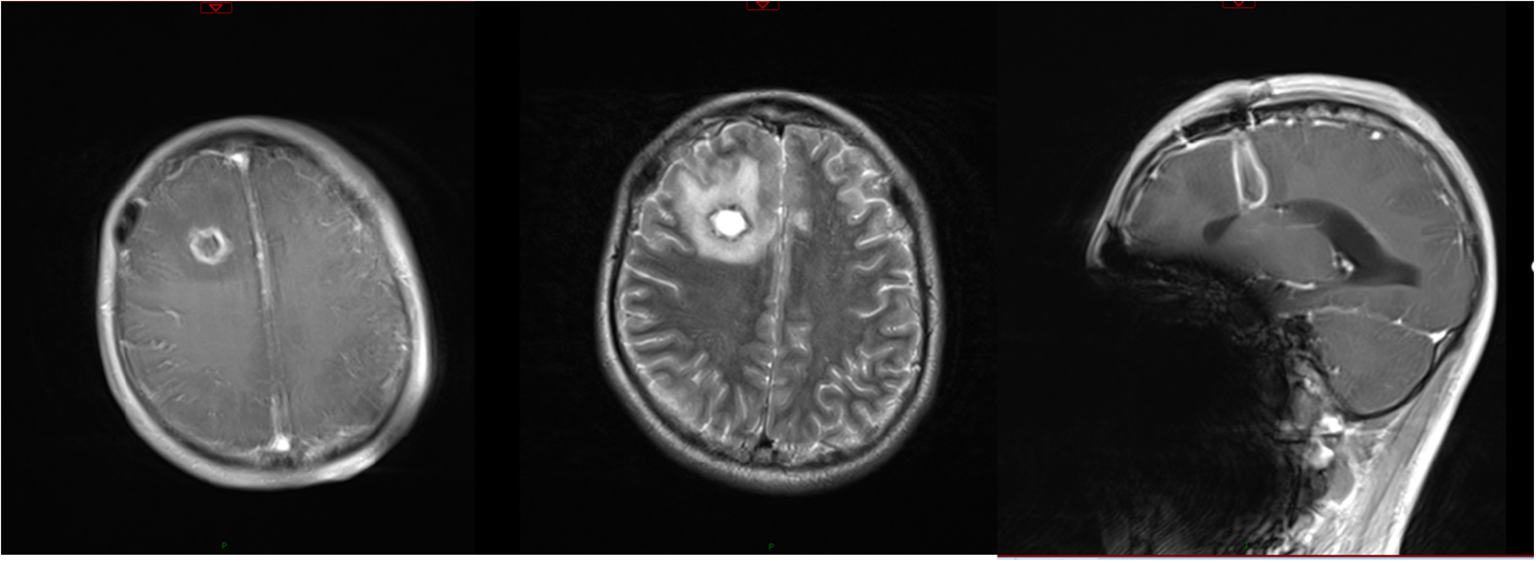
Results of MRI before the new therapeutic regimen. T2 scanning showed that the T2 signal from the interior of the lesion was long, while that from the surrounding cystic wall was short. Enhanced MRI scanning showed significant enhancement along the surgical path. The lesion localized in the surgical area formed a complete cystic wall and was significantly enhanced.

On day 5, the patient had a sudden convulsion of the limbs with loss of consciousness and an upward gaze, which was diagnosed as an epileptic seizure. The seizure was not alleviated by intravenous phenobarbital and valproate, but was eventually controlled by continuous intravenous pumping of midazolam. An urgent routine blood examination found a leukocyte count of 13.9 × 10^9^/L and the neutrophil ratio was 85.4%. Routine CSF and biochemical examination showed a positive Pandy test, leukocyte count increased to 510/mm^3^, microprotein increased to 282 mg/dL, glucose increased to 5.8 mmol/L, and normal chloride (121.8 mmol/L). A head CT scan showed a round cystic-solid lesion in the operative area, with the interior of the lesion being hypointense and the surrounding solid cystic wall being hyperintense. The brain tissue around the lesion was evidently edematous (**Figure 1**). Enhanced MRI of the head showed significant enhancement of all brain parenchyma along the surgical path, and the lesion localized in the surgical area formed a complete cystic wall, which exhibited significant central enhancement (**Figure 2**). Consequently, the child was diagnosed with a brain abscess according to the CT and MRI data. Metagenomic next generation sequencing identified PA. On day 6, analysis of CSF cultures indicated that the pathogen was CRPA (the antimicrobial susceptibility data, which was determined by agar dilution, are shown in **Table 1**). A clinical pharmacist participated in the treatment and suggested adjusting the regimen to intravenous ceftazidime/avibactam (2.5 g, q8h) combined with intrathecal CMS (125,000 U, qd).

**Table 1:** Antimicrobial susceptibility data.

Antibiotics	MIC (mg/L)	Susceptibility
Amikacin	≥64	resistant
Piperacillin/Tazobactam	≥128	resistant
Imipenem	≥8	resistant
Levofloxacin	≥8	resistant
Ceftazidime	32	resistant
Tobramycin	8	resistant
Ciprofloxacin	2	resistant
Cefepime	≥32	resistant
Meropenem	≥8	resistant
Cefoperazone/Sulbactam	≥64	resistant

On day 9, after treatment with the new regimen for 3 days, the child’s body temperature and routine blood results returned to normal, but cervical resistance remained positive. The CSF was pale yellow and the Pandy test was weakly positive. The CSF leukocyte count was reduced to 284/mm^3^, microprotein was reduced to 143 mg/dL, glucose to 2.58 mmol/L, while chloride remained normal at 124.4 mmol/L. The head CT showed that the lesion was smaller and there was reduced edema in the surrounding brain tissue. All of these results suggested that the regimen exerted therapeutic effectiveness.

**Figure 3:**
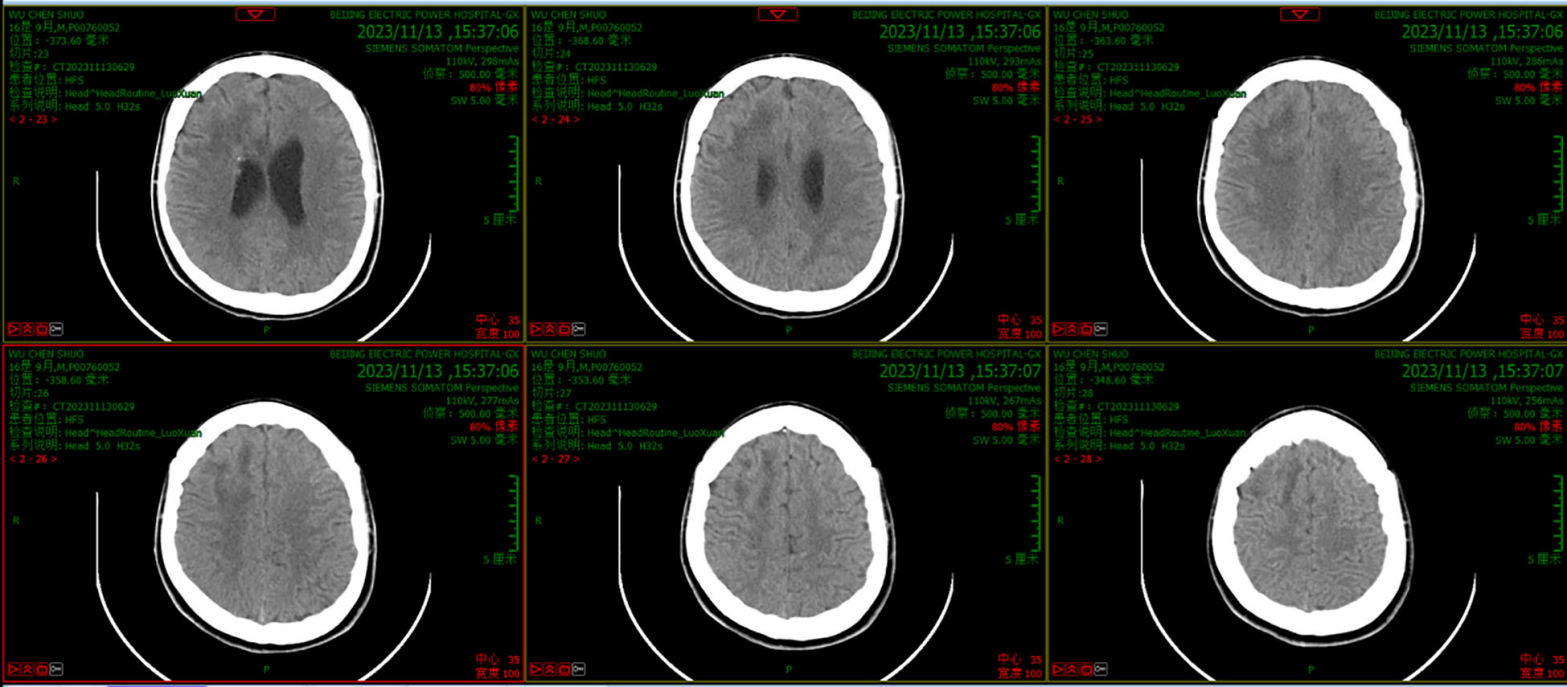
CT scanning results after treatment with the new regimen for 14 days showed that the abscess had disappeared and that there was little edema in the surrounding brain tissue.

**Figure 4:**
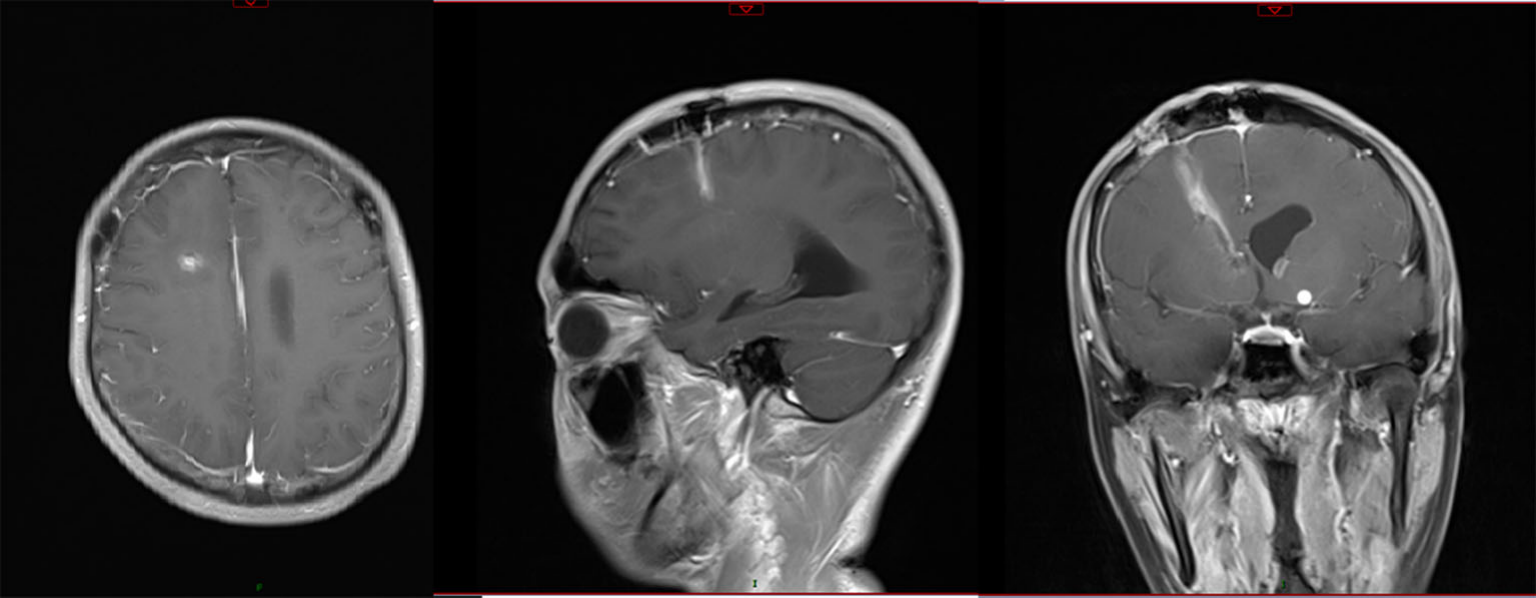
Enhanced MRI scanning after treatment with the new regimen for 14 days showed that the abscess had disappeared, with little enhancement along the surgical path.

**Figure 5:**
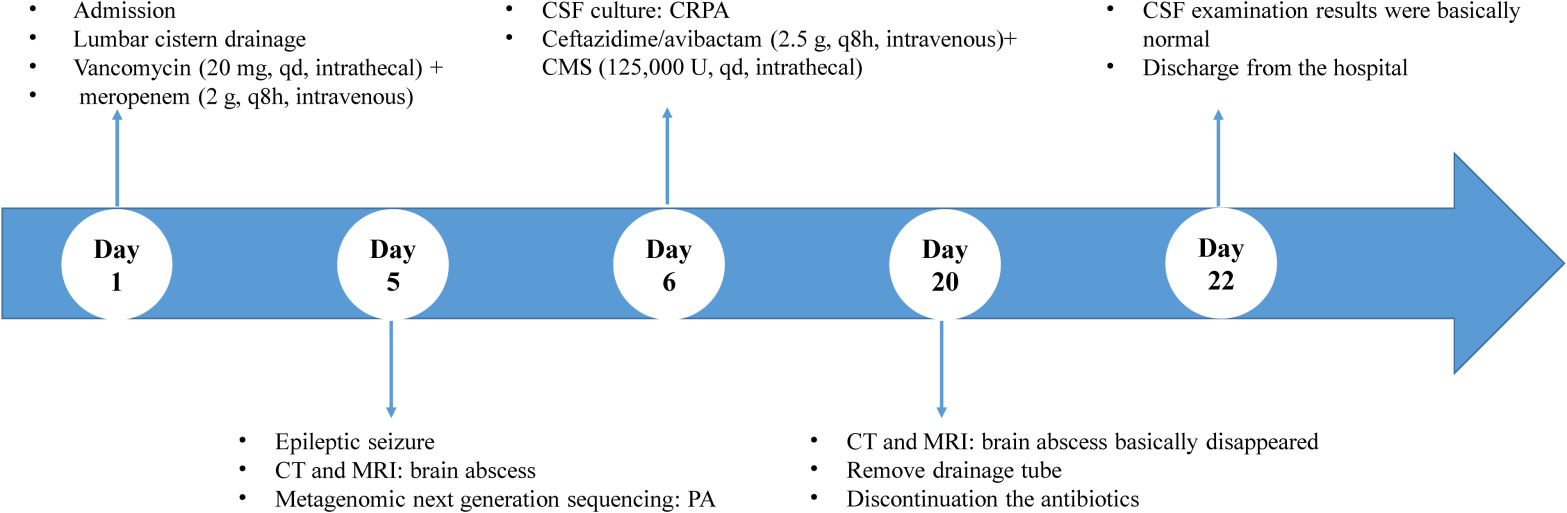
A summary of the clinical treatment process.

On day 20, after treatment with the new regimen for 14 days, the patient’s body temperature and routine blood results remained normal, with negative cervical resistance. The CSF was colorless and clear, and the Pandy test was negative. The CSF leukocyte count decreased to 54/mm^3^, microprotein reduced to 56 mg/dL, while chloride (125.6 mmol/L) and glucose (2.61 mmol/L) were unchanged. A head CT and enhanced MRI showed that the abscess cavity had disappeared (**Figures 3**, **4**). According to these results, the therapeutic regimen was terminated and the lumbar drainage tube was removed. Two days later, the child underwent lumbar puncture again and the CSF examination showed a further reduction of leukocyte count to 28/mm^3^, microprotein was 57 mg/dL, while chloride (126.6 mmol/L) and glucose (3.16 mmol/L) remained normal. Ultimately the child was successfully cured and discharged from the hospital. A summary of the clinical treatment process is shown in **Figure 5**.

## 3 Discussion

Brain abscess is a focal suppurative inflammation of brain tissue with a distinct wall that is associated with a high rate of lethality and disability. Because of the high risk of death in patients with intracranial CRPA-induced infections, the World Health Organization has classified CRPA as a strain that urgently needs new antibiotic treatment as well as innovative and effective therapies (14).

Because there are few reports on CRPA-induced CNSI, the optimal antimicrobial regimen remains unclear. In the present case, the results of antimicrobial sensitivity screening showed that the pathogen was not only resistant to conventional β-lactam/β-lactamase inhibitors (piperacillin/tazobactam, cefoperazone/sulbactam), quinolones and aminoglycosides, but also resistant to imipenem and meropenem (**Table 1**). The carbapenem-resistance mechanisms of PA are complex, but one is its ability to produce multifarious β-lactamases, including extended-spectrum β-lactamases (ESBLs), AmpC β-lactamases, carbapenemases such as Klebsiella pneumoniae carbapenemase (KPC), and oxacillinase-48-like (OXA-48) carbapenemases (15). Avibactam, a novel semisynthetic β-lactamase inhibitor, inhibits the above enzymes to restore the efficacy of ceftazidime against CRE (16). Globally, the susceptibility of CRPA to ceftazidime/avibactam is above 60% (17). Currently, ceftazidime/avibactam has been approved by the US Food and Drug Administration for the treatment of hospital-acquired pneumonia, complicated abdominal infections, complicated urinary tract infections, and associated bacteremia caused by CRE, including CRPA. There are, however, limited clinical data available on the treatment of CRPA-induced CNSI with ceftazidime/avibactam.

To investigate the clinical therapeutic effects of ceftazidime/avibactam against PA-induced CNSI, we conducted a comprehensive review of literature in the PubMed, Medline, and Web of Science databases from their inception to January 2024 for all case reports and series reports on CNSI caused by PA. The search keywords were: *Pseudomonas aeruginosa*, brain abscess, meningitis, ventriculitis, central nervous system infection, and ceftazidime/avibactam. Only seven reports were found that covered the use of ceftazidime/avibactam to treat PA-associated CNSI, among which the pathogens in six cases were CRPA. In one case, drug resistance of the pathogen was not described (**Table 2**).

**Table 2:** Summary of cases of ceftazidime/avibactam in the treatment of *Pseudomonas aeruginosa*-induced CNSI.

Author/ Published Time	Patient Age	Diagnosis	Potential Risk Factors	Strain Characterization	Drug Sensitivity Results of C/A	Treatment Program before C/A	Dosage and course of C/A	Combined Medicine	Prognosis
Yasmin M,2023 (8)	69	meningitis	lung transplantation, ventricular leakage	DTR-PA	Unknow	Ceftazidime + Amikacin	2.5 g, q8h, intravenous, 21 days	CMS 125,000 U, qd, intrathecal injection + CMS 4.5 MU, q12h, intravenous,21 days	clinical cured
Gatti M,2022 (9)	52	meningitis	craniotomy	CRPA	C/A 4 mg/L	Cefepime + Compound Sulfamethoxazole	2.5 g, q6h, intravenous, 23 days	fosfomycin 16g/d (Continuous intravenous pumping),23 days	CSF test turned negative
Gofman N,2018 (10)	32	ventriculitis	cranial removal surgery	PA+CRKP	Ceftazidime 2 mg/L, Avibactam 1 mg/L	Vancomycin, linezolid, ceftriaxone, cefepime, meropenem	2.5 g, q8h, intravenous,42 days	Amikacin 30 mg, qd, intrathecal injection,28 days	CSF test turned negative 3 days later
Zhou Q,2021 (11)	21	acute purulent meningitis	cranial removal surgery	MDR-PA	ceftazidime 8 mg/L	①meropenem 1.5 g, q6h, intravenous +linezolid 0.6 g, q12h, intravenous,10 days. ② CMS 500,000 U, q8h, intravenous +CMS 50,000 U, qd, intracerebroventricular injection, 3 days. ③ CMS 500,000 U, q8h, intravenous + CMS 50,000 U, qd(intracerebroventricular injection)+amikacin 400 mg, q12h, intravenous, 24 days	2.5 g, q8h, intravenous, 14 days	Amikacin 600 mg, q12h, intravenous, 14 days	CSF test turned negative 6 days later
Xipell M,2017 (12)	56	meningitis	renal transplant, sinus debridement	XDR-PA	Unknow	Meropenem 2 g, q8h, intravenous + CMS 2 MU, q8h, intravenous	2.5 g, q8h, intravenous, 30 days	CMS 2 MU, q8h, intravenous	clinical cured
Rodríguez-Núñez O, 2018 (13)	58	suppurative meningitis	renal transplant, maxillofacial surgery	XDR-PA	Unknow	Meropenem + ciprofloxacin, CMS	Undescribe dosage, 38 days	CMS (undescribe dosage intravenous),7 days	survived for 90 days
Almangour TA, 2022 (26)	2	CNSI	ventriculoperitonealshunt	MDR-PA	Ceftazidime 2 mg/L, Avibactam 4 mg/L	vancomycin, ceftazidime, meropenem, amikacin, gentamicin, colistin	450 mg, q8h, intravenous, 21 days	colistin 125,000 U, intraventricular, 14 days	clinical cured

XDR, extensively drug resistant; MDR, multidrug resistant; DTR, difficult-to-treat resistant; CRKP, carbapenem-resistant Klebsiella pneumoniae; C/A, ceftazidime/avibactam; CSF, cerebrospinal fluid.

We found from the seven reports that: (1) the medication regimens were ceftazidime/avibactam combined with another drug, including intrathecal CMS, intravenous or intrathecal aminoglycosides, and intravenous fosfomycin; and (2) the ceftazidime/avibactam concentration in CSF from one case suggested that a standard dose (2.5 g, q8h) could achieve effective therapeutic concentrations (ceftazidime: 21-29 µg/mL, avibactam: 1.42-2.44 µg/mL) (8). Previous studies have demonstrated that ceftazidime effectively penetrated into inflammatory CSF (18), whereas the ability of avibactam to cross the BBB requires further investigation. Yasmin M (8) used astandard dose of ceftazidime/avibactam to treat a patient with CRPA-induced ventriculitis and found that the steady-state concentration ratio of avibactam in CSF compared to serum was in the range of 20% to 38%, which is consistent with that observed in a rabbit meningitis model (19). The percentage of the dosing interval during which ceftazidime/avibactam free drug concentration was above the MIC (% fT > MIC) reached 50% (12). Gatti M reported that if the dose of ceftazidime/avibactam was increased to 2.5 g q6h, the % fT > MIC increased to 100% (9). These results indicate that ceftazidime/avibactam can reach an effective therapeutic concentration for treating CRPA-induced infections in CSF. As drug concentration in brain abscesses is difficult to measure in clinical practice, the drug concentration in CSF is used as a surrogate in the clinic based on the assumption that drugs in CSF can permeate through the brain abscess by continuous exchange with interstitial fluid. However, this exchange between CSF and interstitial fluid barely reaches an equilibrium state (20). Unfortunately, there are no studies on whether an effective therapeutic concentration of ceftazidime/avibactam can be achieved in brain abscesses. Considering that the location of the infected lesion in this patient was deep within the brain parenchyma, we selected ceftazidime/avibactam combined with polymyxins such as CMS to achieve an optimal therapeutic effect.

Polymyxins are antibiotics having cationic cyclic peptide structures that have strong affinity for lipid A of the bacterial extracellular membrane. They are able to competitively replace magnesium and calcium ions of lipopolysaccharide to increase permeability of the bacterial extracellular membrane, resulting in leakage of the cellular contents, and thus exert bactericidal effects (21). Accordingly, polymyxins have excellent antimicrobial activity against most carbapenem-resistant gram-negative bacilli, including CRPA. However, the low penetration of intravenous polymyxins into CSF does not provide an effective bactericidal concentration at conventional doses, and high intravenous doses of polymyxins carry a risk of renal damage (22). The Infectious Disease Society of America recommended ventricular/intrathecal administration of polymyxins to treat intracranial infection, so that polymyxins in the CSF can diffuse and penetrate into the brain abscess and exert their antibacterial effect (20, 23). Currently, polymyxins used in the clinic include CMS, polymyxin B and polymyxin E. Only CMS has been approved for ventricular/intrathecal administration. Because the child patient in this case only underwent lumbar puncture drainage, we adopted intrathecal injection of CMS to treat CRPA-induced intracranial infection.

CMS can remove lipopolysaccharide from the bacterial outer membrane, which facilitates binding of ceftazidime/avibactam to penicillin-binding proteins to inhibit synthesis of bacterial cell wall, thus exerting a synergistic antibacterial effect. Based on the above analysis, we adopted a therapeutic regimen of intravenous ceftazidime/avibactam combined with intrathecal CMS. After 14 days of this combined regimen, the patient’s brain abscess had disappeared and the results of routine CSF and biochemical tests were essentially normal. Ultimately, he was discharged from the hospital successfully without any adverse drug reactions. The difference between our case and other reported cases was that the brain abscess in our case had formed after severe CNSI. Enhanced head MRI showed that the abscess was completely encapsulated. Generally, under these circumstances, surgical abscess resection is required. However, our protocol of intravenous ceftazidime/avibactam combined with intrathecal CMS effectively cured the brain abscess, which avoided the pain of a second craniotomy for the child patient. Therefore, the therapeutic regimen in our case provided a novel therapeutic approach for the treatment of CRPA-induced brain abscess. Recent findings suggested that attenuating cytokine-induced microglial activation, for instance, Th1 and Th17 cytokines in Gram-positive cocci-induced and Th2 cytokine in Gram-negative bacilli-induced CNSI, may contribute to a reduction of the brain abscess and promote the restoration of neurological function (24, 25). The major limitation of this case was that our present study was predominantly focused on the pharmacological rationale behind the bactericidal effectiveness of antibiotics at the lesion site, with no investigation into the possible efficacy in neuroendocrine-immune interaction, which can be undertaken in future research.

In conclusion, according to the experience from our present case, we believe that intravenous ceftazidime/avibactam combined with intrathecal CMS may be an effective therapeutic regimen to treat CRPA-induced CNSI, which is worthy of clinical reference.

## Data Availability

The original contributions presented in the study are included in the article/supplementary material. Further inquiries can be directed to the corresponding authors.

## References

[1] Yang KY Chang WN Ho JT Wang HC Lu CH. Postneurosurgical nosocomial bacterial brain abscess in adults. Infection. (2006) 34:247–51. doi: 10.1007/s15010-006-5607-5 17033747

[2] Brouwer MC Coutinho JM van de Beek D. Clinical characteristics and outcome of brain abscess: systematic review and meta-analysis. Neurology. (2014) 82:806–13. doi: 10.1212/WNL.0000000000000172 24477107

[3] Corsini Campioli C Castillo Almeida NE O'Horo JC Wilson WR Cano E DeSimone DC. Diagnosis, management, and outcomes of brain abscess due to gram-negative versus gram-positive bacteria. Int J Infect Dis. (2022) 115:189–94. doi: 10.1016/j.ijid.2021.12.322 34902581

[4] Tamma PD Aitken SL van Duin D Clancy CJ. Infectious Diseases Society of America 2022 Guidance on the Treatment of Extended-Spectrum β-lactamase Producing Enterobacterales (ESBL-E), Carbapenem-Resistant Enterobacterales (CRE), and *Pseudomonas aeruginosa* with Difficult-to-Treat Resistance (DTR-P. aeruginosa). Clin Infect Dis. (2022) 75:187–212. doi: 10.1093/cid/ciac268 35439291 PMC9890506

[5] Dey R Bishayi B. Ciprofloxacin and dexamethasone in combination attenuate S. aureus induced brain abscess via neuroendocrine-immune interaction of TLR-2 and glucocorticoid receptor leading to behavioral improvement. Int Immunopharmacol. (2021) 97:107695. doi: 10.1016/j.intimp.2021.107695 33962227

[6] Dey R Bishayi B. Ascorbic acid along with ciprofloxacin regulates S. aureus induced microglial inflammatory responses and oxidative stress through TLR-2 and glucocorticoid receptor modulation. Inflammopharmacology. (2022) 30:1303–22. doi: 10.1007/s10787-022-01012-z 35704229

[7] Dey R Bishayi B. Dexamethasone exhibits its anti-inflammatory effects in S. aureus induced microglial inflammation via modulating TLR-2 and glucocorticoid receptor expression. Int Immunopharmacol. (2019) 75:105806. doi: 10.1016/j.intimp.2019.105806 31401378

[8] Yasmin M Nutman A Wang L Marshall S Chen K Wang J. Utilizing ceftazidime/avibactam therapeutic drug monitoring in the treatment of neurosurgical meningitis caused by difficult-to-treat resistant Pseudomonas aeruginosa and KPC-producing enterobacterales. Open Forum Infect Dis. (2023) 10:ofad507. doi: 10.1093/ofid/ofad507 38023540 PMC10661062

[9] Gatti M Virgili G Cojutti PG Gaibani P Conti M Sturiale C. Real-Time Optimization of Pharmacodynamic Target Attainment at Infection Site during Treatment of Post-Neurosurgical Ventriculitis Caused by Carbapenem-Resistant Gram Negatives with Ceftazidime-Avibactam-Based Regimens: A Report of Two Cases. Microorganisms. (2022) 10:154. doi: 10.3390/microorganisms10010154 35056602 PMC8777709

[10] Gofman N To K Whitman M Garcia-Morales E. Successful treatment of ventriculitis caused by *Pseudomonas aeruginosa* and carbapenem-resistant *Klebsiella pneumoniae* with i.v. ceftazidime-avibactam and intrathecal amikacin. Am J Health Syst Pharm. (2018) 75:953–7. doi: 10.2146/ajhp170632 29941534

[11] Zhou Q Wang H Zhan T Yang X Wen L. Successful treatment of Ventriculitis caused by MDR/XDR gram-negative bacillus using ceftazidime/avibactam: case series and literature review. Infect Drug Resist. (2021) 14:1691–701. doi: 10.2147/IDR.S306222 PMC8107005 33981150

[12] Xipell M Bodro M Marco F Losno RA Cardozo C Soriano A. Clinical experience with ceftazidime/avibactam in patients with severe infections, including meningitis and lung abscesses, caused by extensively drug-resistant *Pseudomonas aeruginosa* . Int J Antimicrob Agents. (2017) 49:266–8. doi: 10.1016/j.ijantimicag.2016.11.005 27979500

[13] Rodríguez-Núñez O Ripa M Morata L de la Calle C Cardozo C Fehér C. Evaluation of ceftazidime/avibactam for serious infections due to multidrug-resistant and extensively drug-resistant *Pseudomonas aeruginosa* . J Glob Antimicrob Resist. (2018) 15:136–9. doi: 10.1016/j.jgar.2018.07.010 30036695

[14] acconelli E Carrara E Savoldi A Harbarth S Mendelson M Monnet DL. Discovery, research, and development of new antibiotics: the WHO priority list of antibiotic-resistant bacteria and tuberculosis. Lancet Infect Dis. (2018) 18:318–27. doi: 10.1016/S1473-3099(17)30753-3 29276051

[15] Giovagnorio F De Vito A Madeddu G Parisi SG Geremia N. Resistance in *Pseudomonas aeruginosa*: A narrative review of antibiogram interpretation and emerging treatments. Antibiotics (Basel). (2023) 12:1621. doi: 10.3390/antibiotics12111621 37998823 PMC10669487

[16] Zhen S Wang H Feng S. Update of clinical application in ceftazidime-avibactam for multidrug-resistant Gram-negative bacteria infections. Infection. (2022) 50:1409–23. doi: 10.1007/s15010-022-01876-x 35781869

[17] Kiratisin P Kempf M Stone G Utt E. Ceftazidime-avibactam and comparators against *Pseudomonas aeruginosa* isolates collected globally and in each geographical region between 2017-2020. J Glob Antimicrob Resist. (2023) 34:113–8. doi: 10.1016/j.jgar.2023.06.005 37422001

[18] Tunkel AR Hasbun R Bhimraj A Byers K Kaplan SL Scheld WM. 2017 Infectious diseases society of America's clinical practice guidelines for healthcare-Associated ventriculitis and meningitis. Clin Infect Dis. (2017) 64:e34–65. doi: 10.1093/cid/ciw861 PMC5848239 28203777

[19] Falcone M Paterson D. Spotlight on ceftazidime/avibactam: a new option for MDR Gram-negative infections. J Antimicrob Chemother. (2016) 71:2713–22. doi: 10.1093/jac/dkw239 27432599

[20] Pardridge WM. CSF, blood-brain barrier, and brain drug delivery. Expert Opin Drug Deliv. (2016) 13:963–75. doi: 10.1517/17425247.2016.1171315 27020469

[21] Poirel L Jayol A Nordmann P. Polymyxins: antibacterial activity, susceptibility testing, and resistance mechanisms encoded by plasmids or chromosomes. Clin Microbiol Rev. (2017) 30:557–96. doi: 10.1128/CMR.00064-16 PMC5355641 28275006

[22] Nation RL Rigatto MHP Falci DR Zavascki AP. Polymyxin acute kidney injury: dosing and other strategies to reduce toxicity. Antibiotics (Basel). (2019) 8:24. doi: 10.3390/antibiotics8010024 30875778 PMC6466603

[23] Nance E Pun SH Saigal R Sellers DL. Drug delivery to the central nervous system. Nat Rev Mater. (2022) 7:314–31. doi: 10.1038/s41578-021-00394-w PMC10923597 38464996

[24] Konsman JP. Cytokines in the brain and neuroinflammation: we didn't starve the fire! Pharm (Basel). (2022) 15:140. doi: 10.3390/ph15020140 PMC8878213 35215252

[25] Bajpai A Prasad KN Mishra P Singh AK Gupta RK Ojha BK. Distinct cytokine pattern in response to different bacterial pathogens in human brain abscess. J Neuroimmunol. (2014) 273:96–102. doi: 10.1016/j.jneuroim.2014.05.009 24910026

[26] Almangour TA Alsubaie S Ghonem L Almohaini HA Mohammed Bakheet H Altweijri I. Ceftazidime-avibactam for the treatment of multidrug-resistant Pseudomonas aeruginosa central nervous system infection in pediatric patient: A case report. Pediatr Infect Dis J. (2022) 41:436–8. doi: 10.1097/INF.0000000000003439 34955517

